# Current treatment options for craniofacial hyperhidrosis

**DOI:** 10.1590/1677-5449.200152

**Published:** 2020-11-16

**Authors:** Nelson Wolosker, Carolina Brito Faustino, Marcelo Fiorelli Alexandrino da Silva, José Ribas Milanez de Campos, Paulo Kauffman

**Affiliations:** 1 Hospital Israelita Albert Einstein, São Paulo, SP, Brasil.; 2 Universidade de São Paulo – USP, Faculdade de Medicina, Departamento de Cirurgia Vascular e Endovascular, São Paulo, SP, Brasil.; 3 Universidade de São Paulo – USP, Faculdade de Medicina, Departamento de Cirurgia Torácica, São Paulo, SP, Brasil.

**Keywords:** hyperhidrosis, sympathectomy, oxybutynin, hiperidrose, simpatectomia, oxibutinina

## Abstract

Hyperhidrosis (HH) is characterized by sweating exceeding the amount necessary to meet the thermal regulation and physiological needs of the body. Approximately 9.41% of individuals with HH have craniofacial hyperhidrosis (FH). The present study aims to review the most current data in the literature regarding craniofacial hyperhidrosis, including pathophysiology, diagnosis and clinical presentation, treatment options (clinical and surgical), and outcomes. VATS (videothoracoscopy sympathectomy) is considered the gold standard for definitive treatment of axillary or palmar hyperhidrosis. Recently, several studies have shown the usefulness of clinical treatment with oxybutynin hydrochloride, leading to clinical improvement of HH in more than 70% of users. Both clinical and surgical treatment of craniofacial hyperhidrosis have good results. However, surgical treatment of FH is associated with more complications. Clinical treatment with oxybutynin hydrochloride yields good results and can be the first therapeutic option. When the patient is not satisfied with this treatment and has good clinical conditions, surgical treatment can be used safely.

## Resumo

A hiperidrose (HH) é caracterizada por transpiração além da quantidade necessária para manter a regulação térmica e as necessidades fisiológicas do corpo. Aproximadamente 9,41% dos indivíduos com HH apresentam hiperidrose craniofacial (FH). Este estudo tem como objetivo revisar os dados mais atuais da literatura sobre FH, incluindo fisiopatologia, diagnóstico e apresentação clínica, opções de tratamento (clínico e cirúrgico) e desfechos. A simpatectomia por videotoracoscopia (VATS) é considerada o padrão-ouro para o tratamento definitivo da hiperidrose axilar ou palmar. Recentemente, vários estudos demonstraram a utilidade do tratamento clínico com cloridrato de oxibutinina, que leva à melhora clínica da HH em mais de 70% dos pacientes. O tratamento clínico e o cirúrgico apresentam bons resultados no tratamento da FH. No entanto, o tratamento cirúrgico da FH apresenta mais complicações. O tratamento clínico com cloridrato de oxibutinina fornece bons resultados e pode ser a primeira opção terapêutica. Quando o paciente não está satisfeito com esse tratamento e mostra boas condições clínicas, o tratamento cirúrgico pode ser usado com segurança.

## INTRODUCTION

In a very stressful society, sweating increases as a direct adrenergic response to the sympathetic nervous system and has become a frequent complaint in our daily medical practice.^1^

Hyperhidrosis (HH) is defined as sweating exceeding the necessary amount to meet the body’s thermal regulation and physiological needs. It is characterized by excessive sweating in a specific region due to sweat gland hyperfunctioning, directly affects quality of life, and compromises the patient emotionally, socially, and professionally. HH can be classified as primary or secondary.^1^^,^^2^

Primary HH has no known cause, is usually bilateral and symmetrical, improves during sleep, and worsens in situations of stress, anxiety, fear, and nervousness. It may have a related genetic predisposition as evidenced by its familial transmission through dominant autosomal genes. Secondary HH is due to an organic cause (such as endocrine and neurological diseases, substance use, menopause, neoplasia, or infections). In these cases, it is always necessary to look for the underlying cause and to plan the appropriate treatment.^3^

HH occurs in 2.8% of the US population and rates are probably higher in tropical countries.^4^ The incidence of craniofacial hyperhidrosis (FH) is unclear in the literature, but Wolosker et al. identified the following primary sites of primary HH in a sample of 1657 patients: 48.34% palmar hyperhidrosis, 35.96% axillary hyperhidrosis, and 9.41% craniofacial hyperhidrosis (FH).^5^ There are therefore probably more than 800,000 individuals with FH in the United States today.

According to the aforementioned sample, a majority of FH patients (55.1%) are female, although 44.9% are males, and some studies show a predominance of males. The mean age is over 40 years of age; onset is usually in adulthood; and it is not an uncommon complaint among menopausal women.^5^ It worsens with age and, like hyperhidrosis of any other site, worsens in overweight and obese patients.^6^

FH can occur on the entire face but especially on the forehead (where the highest concentration of eccrine sweat glands is found) and on the upper lip. It is predominantly diurnal and almost never occurs at night.

Current treatment approaches for FH include videothoracoscopy sympathectomy (VATS), topical application of aluminum chloride, topical use of an anticholinergic agent such as glycopyrrolate, oral anticholinergic medication such as oxybutynin, and intradermal injections of botulinum toxin A. Efficacy is usually evaluated in terms of reported improvement in quality of life (using specific questionnaires) and additional side effects.^2^^-^6

This article will cover the following information about FH: physiopathology, diagnosis and clinical presentation, clinical treatment, surgical treatment and treatment failure/relapse.

## PHYSIOPATHOLOGY

The human body has approximately 4 million sweat glands, of which 3 million are eccrine and the remaining are apocrine.^7^ The eccrine sweat glands are epidermal appendages innervated by sympathetic nervous system cholinergic fibers, whose main function is to produce sweat, an odorless and colorless liquid responsible for the regulation of body temperature. They are present on the entire surface of the body, predominantly in the palmar, plantar, craniofacial, and axillary regions, and are less abundant in the thorax and dorsum.^8^ Each gland is composed of a secretory portion located in the dermis and associated with a rich capillary plexus and a long duct that connects that portion to the epidermis. Entry of extracellular calcium into the secretory cell is the essential mechanism for controlling stimulation and activation of ions and water (eccrine secretion).^8^^-^10

**Figure 1:**
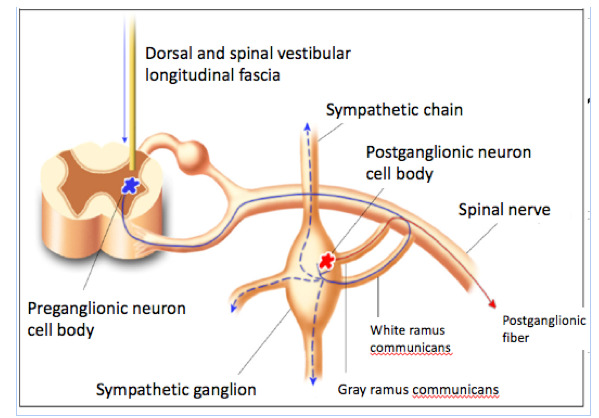
Nerve impulse pathway to the sympathetic ganglion.

The apocrine sweat glands, also known as scent glands, are limited to the axillary and urogenital regions. They do not participate in localized hyperhidrosis, and their activation is regulated by hormonal processes.^7^ No histopathological changes were detected in patients with primary HH, not even an increase in the number of sweat glands. However, primary HH may constitute a complex dysfunction of the autonomic nervous system involving the sympathetic and parasympathetic pathways.^7^ The sweat thermoregulatory center is found in the hypothalamus, more precisely in the preoptic region.^11^ The sympathetic motor pathway consists of three neurons, as follows. The first has its cellular body located in nerve and vasomotor centers, and its axon descends through the dorsal and spinal vestibular longitudinal fascia of the spinal cord, establishing synapses with the cellular body of the second neuron. The second neuron, also known as the preganglionic neuron, is located in the medial-lateral column of the medullary gray matter (Clarke's column), which extends from the first thoracic segment to the second lumbar segment. Its axon (preganglionic fiber) leaves the medulla along with the ventral roots of the spinal nerves, goes through the white ramus communicans, and ends in the paravertebral sympathetic trunk ganglia, where it establishes synapses with the cellular body of the third neuron (postganglionic neuron). The axon of this neuron (postganglionic fiber) leaves the sympathetic chain through the gray ramus communicans, enters the spinal nerve and is peripherally distributed to the sweat glands (Figure 1).^7^^,^^11^

The sympathetic ganglia are arranged longitudinally on each side of the spinal cord and are connected by the interganglionic segments. There are three cervical ganglia (upper, middle, and lower) and 10 to 12 thoracic, 2 to 5 lumbar, 4 to 5 sacral, and 1 coccygeal ganglia. The craniofacial region is innervated by sudomotor (preganglionic) fibers, which, in most cases, come from the first to the fifth thoracic medullary segments; the upper limbs, from the second to the eighth thoracic segments, and the lower limbs, from the tenth thoracic segment to the second lumbar segment.^12^^,^^13^

**Figure 2:**
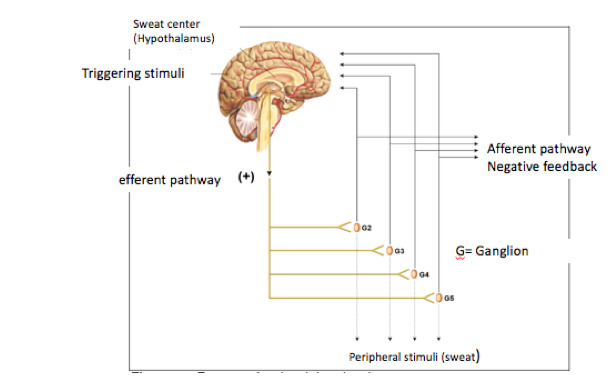
Functional schematics of the sweat pathways.

Acetylcholine is the chemical mediator released at the neurogland junction of postganglionic fibers, in contrast with the terminal nerves of these fibers in most sectors, where the chemical mediator is noradrenaline. Activating stimuli (e.g., anxiety, stress, high temperature environments, and physical exercise) activate the preoptic region of the hypothalamus, which releases acetylcholine at the neurogland junction through sympathetic stimulation. This causes an increase in the sweat response through stimuli transmitted by the efferent pathways to the sympathetic ganglia. After triggering sweating, these stimuli return to the hypothalamus through the afferent pathways (negative feedback) (Figure 2).^14^

The balance between these pathways (efferent and afferent) maintains homeostasis of the body, but this system is exaggerated in patients with HP.^14^

## DIAGNOSIS AND CLINICAL PRESENTATION

The diagnosis of primary hyperhidrosis is mainly clinical and is based on patient history and physical examination. In FH, sweat is visible on the forehead and face.^15^

Criteria exist for identifying primary hyperhidrosis and to facilitate correct diagnosis.

Criteria for diagnosis:

Visible, excessive, and localized sweat lasting at least 6 months, with no apparent cause and having at least two of the following features:

• Bilateral and symmetrical sweating;

• Frequency of at least one episode per week;

• Impairment of daily activities;

• Onset before 25 years of age;

• With family history;

• No sweating during sleep.

Body thermoregulation depends on the sweating mechanism. In some physiological situations, it is possible to observe sweat gland hyperactivity, since it occurs during and after physical exercise in obese people and during menopause. The first step is to differentiate between primary (focal) and secondary hyperhidrosis. Secondary hyperhidrosis is generally associated with underlying diseases or conditions, such as infections, neoplasms or hormonal disorders. In contrast with secondary hyperhidrosis, primary hyperhidrosis occurs in healthy individuals (Table 1).^3^

**Table 1:** Main causes of hyperhidrosis.

**Types of hyperhidrosis**	**Commonly associated diseases and conditions**
Primary hyperhidrosis	Idiopathic (focal) hyperhidrosis
	Gustatory hyperhidrosis (Frey’s syndrome)
Secondary hyperhidrosis	Endocrine: hyperthyroidism, hyperpituitarism, diabetes mellitus, menopause, pregnancy, pheochromocytoma, carcinoid syndrome, and acromegaly
	Neurological: Parkinson's disease, spinal cord injury, and stroke
	Neoplastic: Hodgkin's disease and myeloproliferative diseases
	Infectious: tuberculosis and septicemia
	Drugs: fluoxetine, venlafaxine, doxepin
	Toxicity: alcoholism and illicit substance abuse

It is not the purpose of this paper to discuss gustatory hyperhidrosis (Frey’s syndrome).

## CLINICAL TREATMENT

At our service, we have 20 years of experience with hyperhidrosis and have followed-up more than 2,400 patients treated with VATS and 1,500 patients treated with oral oxybutynin.^2^^,^^6^

Over the last ten years, we have conducted several studies showing the usefulness and safety of treatment with oxybutynin hydrochloride, an anticholinergic, that leads to clinical improvement of HH in more than 70% of users.^16^ Since then, we have used this medication as the first line treatment. However, in patients who do not respond adequately to this medication,^17^ VATS is considered the gold standard for definitive treatment of hyperhidrosis, having a considerable impact and improving the quality of life, with good results.^18^^-^23

Oxybutynin can be prescribed to children and adults, but angle-closure glaucoma is a contraindication. At the initial consultation, oxybutynin is administered according to the following protocol: during the first week, patients take 2.5 mg oxybutynin once daily at bedtime. Between days 8 and 21, they take 2.5 mg twice daily. From day 22 onwards, they take 5 mg twice daily. Pediatric patients weighing less than 40 kg follow the same treatment up to a maximum dose of 5 mg/day.^19^^-^22^,^^24^^,^^25^ The doses of the drug are adjusted on a case-by-case basis, balancing between a better response and lower side effects, with a minimum dose of 5 mg/day and a maximum dose of 15 mg/day distributed according to the intensity of the patients' symptoms throughout the day, with a minimum interval between doses of 6 hours and a maximum interval of 24 hours. The main side effects are dry mouth and headache. The efficacy of treatment is assessed at each visit using specific questionnaires covering quality of life and the impact of HH on daily activities. The most recent retrospective analysis by our group, which evaluated 1,685 patients with HH including 141 with FH, showed improvement in the quality of life in 85.8% of HH patients. Surgical treatment is discussed with patients who still have very poor quality of life despite treatment with oxybutynin.^24^^-^26

Topical treatment for hyperhidrosis can be administered using antiperspirant substances that block the eccrine sweat gland duct or cause secretory cell atrophy, such as 20% aluminum hydroxide. The literature shows it is safe to use for axillary and palmar regions, but there is scant data regarding its use on the face, probably due to the risk of irritability.^3^

Botulinum toxin is a neurotoxin derived from the anaerobic bacterium *Clostridium botulinum*. It acts on the presynaptic terminal of a neuron at the neuromuscular junction, preventing release of acetylcholine and causing a temporary denervation with loss or decrease of the activity of the target organ. The therapeutic effect is temporary, and treatment should be repeated at regular intervals. Durability of the treatment is variable and depends on the individual, but lasts 3 months on average depending on the characteristics of each patient. Use for hyperhidrosis is well-documented in the literature, but publications on FH are scarce.^3^^,^^27^^,^^28^

Karlqvist et al. proposed the use of botulinum toxin type B for more aggressive treatment of craniofacial hyperhidrosis in 38 patients; their rationale for use of this toxin was that it would have a less pronounced effect on motor neurons and would cause less muscle weakness, an important factor when treating hyperhidrosis.^27^ They administered 5 U intradermally to points every 15 mm all over the face, covering the entire hyperhidrosis area, especially the forehead. They claim to have used between 110 and 2300 U depending on the case. According to this publication, 87% of the patients were satisfied with the administration and the improvement in the quality of life, 18% presented stiffness in the forehead, and 18% showed changes affecting the eyebrows. Cabreus et al. studied postmenopausal craniofacial hyperhidrosis treated with botulinum toxin type B. They studied a subgroup of eight postmenopausal patients participating in a randomized controlled trial of botulinum toxin type B treatment for craniofacial hyperhidrosis. The results indicated that botulinum toxin type B seems to be a safe and effective treatment in postmenopausal craniofacial hyperhidrosis.^3^^,^^28^

Use of botulinum toxin is particularly indicated for areas of localized sweating. Administration should be as superficial as possible and preferably intradermal, to form papules. We prefer to use a dilution of 100 U of botulinum toxin in 1.0 ml of saline solution or 500 U Dysport® in 1.6 ml of saline. Knowledge of the facial muscles is crucial when treating hyperhidrosis, as toxin is always dispersed to the adjacent musculature.

In FH of the upper lip: We divide the upper lip into two halves and apply 1 U botulinum toxin to 3 points in each half at 0.5 cm from the transition of the lip and the skin, 0.5 cm from the philtrum, and 0.5 cm between each point. Each case should be treated individually and analyzed separately, but we consider that this dose is the safest minimum dose to treat hyperhidrosis of the upper lip, with a lower risk of side effects in the region, which includes the orbicularis oris muscle (decreased force of bite; difficulty in pronouncing the letters B and V; and difficulty using a straw, whistling, and mouthwashing). The patient should always be informed of these possible side effects, but they are usually transient and last approximately 2 weeks. In contrast, the patient will have reduced upper perioral wrinkles. Treatment of hyperhidrosis with botulinum toxin has good esthetic results and side effects, which should be explained to the patient as in any other treatment. The durability of the treatment is variable and depends on the individual but lasts 3 months on average depending on the characteristics of the patient.

In frontal FH: In the frontal region, administration of botulinum toxin for treatment of hyperhidrosis is more diffuse than for aesthetic applications and more comfortable than in the case of upper lip administration. We usually distribute points every 1 cm across the forehead up to the hair, except for an area 1 cm from the line of the eyebrow. We proceed in the same manner, administering1 U of botulinum toxin at each point. We generally conduct an aesthetic assessment of the patient to judge the need for treatment of the glabella and, if necessary, proceed with the treatment to better harmonize the upper 1/3 of the face. It is essential to explain to the patient that “spreading” botulinum toxin on the entire forehead will have a “freezing” effect on the expression of the upper 1/3 of the face, specifically on the eyebrows. In contrast, the patient will benefit from smoothing of frown lines on the forehead, which generally pleases the patient. Although we use a reduced dose of 1 U per point and the application is administered intradermally, the frontal muscle is very close to the skin and diffusion will certainly occur. In some cases, we treat the patient by focusing on the aesthetics, distributing the points so that the eyebrows acquire a harmonious appearance, even if the patient continues to partially sweat from the forehead afterwards.

All patients treated with botulinum toxin are reviewed after 15 days, and supplementary points are administered to areas where it is deemed necessary (continued sweating and minimal impairment of facial expressions). We usually apply a local topical anesthetic compound 30 minutes prior to administration and proceed with cleansing with chlorhexidine-alcohol. We use a 50 U needle syringe with a 30 and ½G needle.

Currently, in the authors’ daily practice, botulinum toxin cannot always be used, as there is always some change of the facial expression, even with extreme care. Additionally, botulinum toxin is a localized treatment for craniofacial hyperhidrosis, which is not always consistent with a patient's history, since they generally complain of much more extensive hyperhidrosis on the face. In some cases, the authors proceed with combined treatment with botulinum toxin and oxybutynin, which has excellent results and requires smaller doses of botulinum toxin.

## SURGICAL TREATMENT

Our group has been performing surgical treatment with VATS for 20 years.^2^^,^^6^^,^^17^ Sympathectomy is defined as ablation on the ribs and on the surface between the chosen ganglia. Easy visualization of the chain, a small scar, less discomfort for the patient, and absence of Horner’s syndrome are the main advantages of the technique compared to nonthoracoscopic techniques. The technique was further improved by Byrne et al. by adoption of selective orotracheal intubation and use of a double-lumen catheter, no routine chest drainage, and use of two incisions, which, by decreasing the size of both, causes less postoperative pain.^12^ In our country, this technique was first used in the 1990s, when the vascular surgery team at the Clinical Hospital of the Medical School of the University of São Paulo still performed resection of the sympathetic chain for HH via a supraclavicular approach. It is associated with high rates of therapeutic success, and there is significant improvement in sweating, reducing the discomfort and embarrassment caused to patients and thus solving their psychosocial and professional problems, as well as improving their quality of life.^2^^,^^6^^,^^17^ With technological development of the materials employed, the surgical technique improved, increasing the safety of the procedure, and, consequently, reducing its morbidity. Some advances include introduction of video lenses with lateral vision (30°), reduction of the diameter of the videothoracoscopy apparatus, and development of specific introducers for the thorax and of the harmonic scalpel. The ultrasonic vibration of the harmonic scalpel allows one to section the sympathetic chain by vaporization, without a significant increase in temperature or transmission of electric current.

Technical development of the surgery led to an increase in the number of patients undergoing sympathectomy, but compensatory hyperhidrosis was more frequently observed. Compensatory HH is defined as a change in the quantitative distribution of sweat in response to external heat, with a compensatory increase of sweating in nondenervated body regions (lumbar, abdominal, and lower limbs) to maintain body thermoregulation. Therefore, it only occurs after approaching the sympathetic chain at the thoracic level. Since the areas of the head, neck, armpits and hands are responsible for the greatest dissipation of body heat by sweating, sympathetic denervation results in increased sweating in other body segments, particularly at higher temperatures and in situations of physical exercise and stress. The first description of compensatory hyperhidrosis was published by Adson in 1935, who found that, after sympathectomy, there was an increase in the amount of sweat in areas of the skin that were not affected by the surgery to maintain a normal body temperature.^9^

There is no established cause or mechanism to explain the onset of compensatory sweating, unlike other complications, such as gustatory sweating, which is triggered by food and emotions. Some authors believe that its appearance is related to a mechanism of compensatory response to temperature regulation, since it is often observed in environments with higher temperatures. Although variable, the incidence of CH can be close to 100% in several studies in the literature, though there is a lower incidence when sympathetic resection is more limited and conducted at a lower point. Despite its high incidence, it should be noted that only a small percentage have the most severe symptoms.^11^^,^^14^^,^^16^ In patients with severe CH, the symptoms become uncomfortable, cause embarrassment and impair daily activities, as well as interfering with work, leisure, and social activities. These patients need to change their clothes more than once a day (and therefore need to take extra clothes with them), often avoid wearing colored clothing, and usually seek supplementary treatment. Several studies have attempted to identify predictive factors of severe compensatory hyperhidrosis, since affected patients may have worse clinical status and quality of life postoperatively, making them regret having had the surgery.^29^^,^^30^

The indication of sympathectomy level has changed over the years as the technique has evolved, as has been observed in the literature and based on our own experience. The approach is defined by the patient's most uncomfortable area of complaint, and T2 is the most appropriate level for FH. It has been shown that the higher the level of the VATS ganglia resection, the greater the incidence of severe CH, which is also associated with resection of more than one ganglion level in the same operation.^31^ Additionally, we observed a higher incidence of Horner's syndrome after higher resections. Obesity is also associated with a higher incidence of severe CH, and therefore at our service we only operate on patients with BMI lower than 25 kg/cm. In our case series of 2431 patients operated, 1.7% of whom had FH, there was an improvement in quality of life in 80.6% of patients reporting FH as the main site of primary HH. Severe compensatory HH occurred in 22.1% of this sample.^32^^-^34

**Figure 3:**
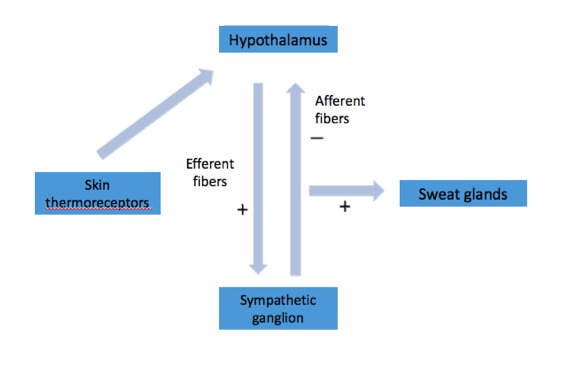
Mechanism of hypothalamic control of sweating.

**Figure 4:**
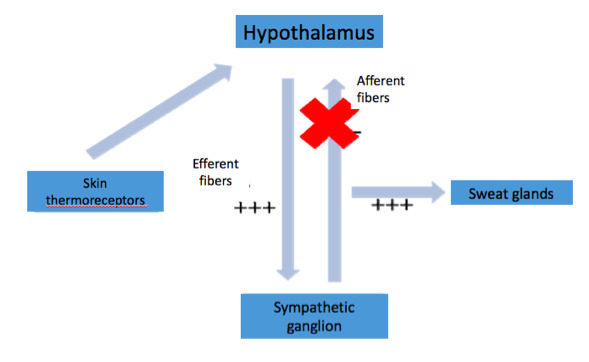
Mechanism of compensatory hyperhidrosis after sympathectomy.

Before sympathectomy, temperature influences the thermoreceptors in the skin, triggering a response from the thermoregulatory center located in the hypothalamus.^31^^,^^35^ The sympathetic tone efferent to the hypothalamus would then induce sweating through synapses in the sympathetic ganglia, triggering a negative sympathetic stimulus afferent to the hypothalamus after the synapse (Figure 3). In cases of sympathectomy at the T2 or T3 level, the efferent sympathetic stimulus is amplified, because there is interruption of the afferent fibers into the hypothalamus, and there is no sympathetic negative stimulus. Since the amplified sympathetic stimulus does not reach sympathectomized areas, sweating occurs in other parts of the body (Figure 4). In cases of sympathectomy at the T4 level, most of the afferent fibers responsible for the sympathetic negative stimulus are preserved, and thus severe compensatory sweating does not occur. Therefore, compensatory sweating is apparently a reflex mechanism mediated by the hypothalamus and is not fully understood.^36^^,^^37^

The best approach to CH would be avoid causing it in the first place. Indications for treatment of hyperhidrosis should be cautious, and the patient should be informed about the results of surgery, its complications, and the fact that a perfect surgical technique is not currently available. Adequate and detailed information will increase postoperative satisfaction and is therefore an essential part of treatment. Predictive factors for compensatory hyperhidrosis include resection at higher levels and higher body mass index.^34^^,^^38^

**Figure 5:**
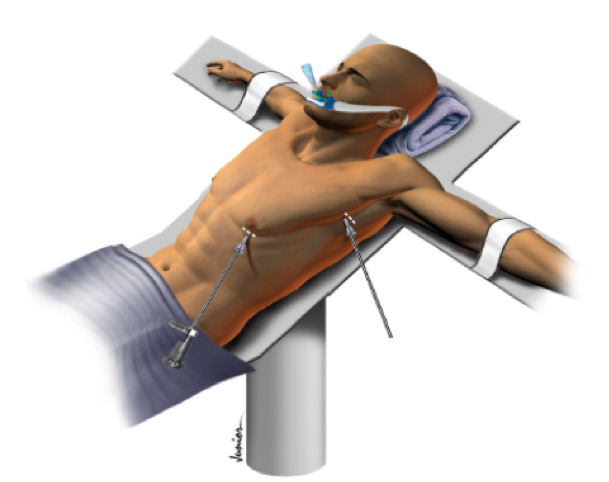
Position of the patient in the operating room.

Regarding the surgical technique, in general terms, surgery is performed with the patient in a semi-Fowler’s position at 45° to the floor. Two small incisions of approximately 1.0 cm are made in each hemithorax. The pleural cavity is accessed through the incision made in the 4th intercostal space, at the anterior axillary line, through which a 5 mm and 30-degree video laparoscope is introduced. The second incision is made in the second intercostal space, at the middle axillary line, for insertion of the endoscopic scalpel (Figure 5).

**Figure 6:**
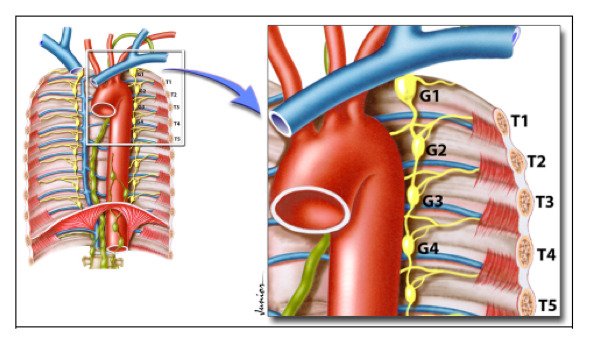
Location of the sympathetic ganglia. G: sympathetic ganglia. T: rib.

After identification of the sympathetic chain, the ganglion is isolated, always starting from the medial costal pleura, following the lateral costal pleura, and ending with complete dissection of the sympathetic chain and the ganglion. The sympathetic chain is resected on the respective costal arches. At the end of this step, the sympathetic chain segment located between the corresponding costal arches, including the target ganglion, is electrocauterized (Figure 6).^39^^,^^40^

During the sympathectomy, all patients are kept temporarily in apnea or low flow ventilation. At the end of the surgery, the residual pneumothorax is aspirated through a No. 16 nasogastric tube and pulmonary expansion is followed by video. The incisions are closed with intradermal sutures using 4-0 monofilament suture threads (monocryl). Anatomically, the sympathectomy performed on the right side of the chest is slightly more laborious compared to the left side because of the higher number of large caliber veins along the thoracic sympathetic chain and its more superior branches, demanding greater care from the surgeon when dissecting. Patients are usually extubated without difficulty in the operating room. After awakening from anesthesia, they are referred for anesthetic recovery and then to their room. To ensure complete pulmonary re-expansion, chest radiographs are routinely performed shortly after the surgery.

Regarding other possible complications, death is very rare in patients submitted to video-assisted thoracic sympathectomy. Until 2003, only eight deaths had been reported in the literature; four occurred due to massive intraoperative hemorrhage, three due to problems related to the anesthetic technique, and one with no apparent cause. Presence of pleural adhesions is another possible complication and may be related to pleuropulmonary diseases during childhood in patients submitted to sympathectomy. This is because the prevalence of acute respiratory infections in our country is one of the main public health problems in children younger than five years, responsible for approximately 20 to 40% of pediatric service consultations. A study by Baumgartner and Youn reported pleural adhesions in 28 (9.1%) of the 309 patients submitted to video-assisted sympathectomy. These authors observed six surgical failures (1.9%) due to intense pleuropulmonary adhesions on the sympathetic chain and the ganglion, impairing their identification and limiting the surgery.^41^

Intercostal neuralgia and pneumothorax have been reported as the main complications of video-assisted thoracic sympathectomy. Several authors have reported other possible complications, such as chylothorax, hemothorax, hemopneumothorax, pulmonary atelectasis, and pleural effusion.

Nowadays, Horner's syndrome has become an uncommon postoperative complication due to the use of the video system and the vast experience of surgeons. The following clinical signs are present in this syndrome: eyelid ptosis, miosis, facial anhidrosis, and sometimes enophthalmia, as described by Freiderich Horner in 1986. Its occurrence is limited to cases of indirect damage of the ganglion by heat diffusion or excessive traction of the sympathetic chain, which was more frequent when the intervention was performed at G2.^42^

Our group’s most recent analysis, covering 2,430 patients operated over 20 years, showed that 1.7% of the patients had FH. In the postoperative period, we observed improvement in quality of life in more than 90% of the patients. Specifically, in FH, 80.6% of the patients submitted to VATS reported clinical improvement at the HH site. Severe CH occurred in 22.1% of this sample. Postoperative Horner's syndrome (HS) is rare, but is found in almost all reports, and is caused by direct or indirect damage to T1 by current diffusion or excessive traction on the chain during dissection or ablation. In our series we had 0.5% patients with transient and 0.5% patients with definitive unilateral HS.^43^^-^45

## FOLLOW-UP

At outpatient follow-ups, pre and postoperative quality of life and pre and postoxybutynin use is assessed. To quantify quality of life, a protocol described by Amir et al. and adapted by De Campos et al. was used that comprises 20 questions divided into four domains (functional-social, personal, emotional, and special conditions).^44^^,^^45^ During scheduled evaluations, the patients are classified into five levels of satisfaction obtained by the sum of the points from the questionnaire. The questionnaire is administered preoperatively and at scheduled follow-up consultations including the 1st week, 1st month, 6th month, and 12th month. It is completed by the patient without intervention by the evaluator (Appendix A).

We also evaluate the efficacy of treatment using the HH Disease Severity Scale (HDSS). This scale consists of a simple and straightforward question with four available answers (grades 1 to 4) related to patient tolerance to the symptoms of primary HH and the negative impacts of body sweating on their daily activities. Patients are asked to indicate a specific score for each HH site. For data evaluation purposes, the analysis generates a delta for HDSS improvement. For example: Pretreatment (week 0), minus posttreatment (week of return to the outpatient clinic) =delta. Thus, delta=0 or 1 is considered as no improvement; delta=2 is considered a slight improvement, and delta=3 is considered good improvement.

## TREATMENT FAILURE/RELAPSE

Studies in the literature show that failures in sympathetic denervation contribute to relapse of localized hyperhidrosis. According to Kim et al., the main causes of surgical procedure failure are incomplete resection of the sympathetic chain or ganglion, surgery at an inappropriate site, sliding of the metal clip, and partial regeneration of the sympathetic chain.^46^

Other factors that could contribute to relapse of hyperhidrosis are related to anatomical variations in the sympathetic nervous system. In most individuals, sympathetic innervation of upper limb sweat glands largely originates below the fourth thoracic sympathetic ganglion, but it is possible that there is a more significant contribution of cranially located ganglia, specifically G3 and G2, in a few cases. This would explain not only the immediate surgical procedure failures, but also relapse of hyperhidrosis over the medium term. A simple sympathectomy, that is, resection of the sympathetic chain on the costal arches, could make regeneration of the sympathetic chain more likely by leaving the stumps of the resected sympathetic chain very close to each other.

## EMERGING THERAPEUTIC MODALITY

Another treatment modality that has been described recently for treatment of these patients is sphenopalatine block. The first randomized trial that revealed effects on craniofacial hyperhidrosis from blocking the sphenopalatine ganglion (SPG) with lidocaine was described by Lehrer et al.^47^ Patients with FH or flushing (n=25) were randomized at a ratio of 1:3 into one of two groups: 1) endoscopic application of topical lidocaine over the SPG (TL; n=7); or 2) endoscopic injection of lidocaine into the SPG (IL; n=18). FH was scored with a Visual Analogue Scale (VAS). At baseline, groups reported similar FH VAS (TL: 89.3 plus or minus 17.5mm; IL: 85.7 plus or minus 22.1mm). After 6 months, the least squares mean of FH VAS in the IL was -38.1 (-47.3 to -28.9) compared to TL 1.9 (-12.2 to 15.9). Therefore, the patients included in the study showed significant clinical improvement after injection, lasting 6 months.^47^

## CONCLUSION

FH is a common disease and has a significant impact on patients’ quality of life. Clinical treatment with oxybutynin hydrochloride yields good results and is the first therapeutic option. When the patient is not satisfied with this treatment and has good clinical conditions, surgical treatment can be used safely, but there is a high risk of compensatory hyperhidrosis, and this risk should be explained to patients to enable them to make an informed decision.

## References

[1] Cameron AEP (2016). Selecting the right patient for surgical treatment of hyperhidrosis. Thorac Surg Clin.

[2] Wolosker N, Campos JRM, Kauffman P, Oliveira LA, Munia MAS, Jatene FB (2012). Evaluation of quality of life over time among 453 patients with hyperhidrosis submitted to endoscopic thoracic sympathectomy. J Vasc Surg.

[3] Hornberger J, Grimes K, Naumann M (2004). Recognition, diagnosis, and treatment of primary focal hyperhidrosis. J Am Acad Dermatol.

[4] Cerfolio RJ, Campos JRM, Bryant AS (2011). The Society of Thoracic Surgeons Expert Consensus for the Surgical Treatment of Hyperhidrosis. Ann Thorac Surg.

[5] Estevan FA, Wolosker MB, Wolosker N, Puech-Leão P (2017). Epidemiologic analysis of prevalence of the hyperhidrosis. An Bras Dermatol.

[6] Leiderman DBD, Campos JRM, Kauffman P (2018). The relation between age and outcomes of thoracic sympathectomy for hyperhidrosis: the older the better. J Thorac Cardiovasc Surg.

[7] Sato K, Kang WH, Saga K, Sato KT (1989). Biology of sweat glands and their disorders. I. Normal sweat gland function. J Am Acad Dermatol.

[8] Moura NB, Neves-Pereira JC, Oliveira FR (2013). Expression of acetylcholine and its receptor in human sympathetic ganglia in primary hyperhidrosis. Ann Thorac Surg.

[9] Adson AW (1935). Essential hyperhidrosis cured by sympathetic ganglionectomy and trunk resection. Arch Surg. American Medical Association..

[10] Adar R, Kurchin A, Zweig A, Mozes M (1977). Palmar hyperhidrosis and its surgical treatment: a report of 100 cases. Ann Surg.

[11] Lyra RM, Campos JRM, Kang DWW (2008). Guidelines for the prevention, diagnosis and treatment of compensatory hyperhidrosis. J Bras Pneumol.

[12] Byrne J, Walsh TN, Hederman WP (1990). Endoscopic transthoracic electrocautery of the sympathetic chain for palmar and axillary hyperhidrosis. Br J Surg.

[13] Wenzel FG, Horn TD (1998). Nonneoplastic disorders of the eccrine glands. J Am Acad Dermatol.

[14] Chou SH, Kao EL, Lin CC, Chang YT, Huang MF (2006). The importance of classification in sympathetic surgery and a proposed mechanism for compensatory hyperhidrosis: experience with 464 cases. Surg Endosc.

[15] Haider A, Solish N (2005). Focal hyperhidrosis: diagnosis and management. CMAJ.

[16] Teivelis MP, Wolosker N, Krutman M, Campos JRM, Kauffman P, Puech-Leão P (2014). Compensatory Hyperhidrosis: Results of Pharmacologic Treatment With Oxybutynin. Ann Thorac Surg.

[17] Lembrança L, Wolosker N, Campos JRM, Kauffman P, Teivelis MP, Puech-Leão P (2017). Videothoracoscopic Sympathectomy Results after Oxybutynin Chloride Treatment Failure. Ann Vasc Surg.

[18] Wolosker N, Campos JRM, Kauffman P, Yazbek G, Neves S, Puech-Leão P (2013). Use of oxybutynin for treating plantar hyperhidrosis. Int J Dermatol.

[19] Wolosker N, Schvartsman C, Krutman M (2014). Efficacy and quality of life outcomes of oxybutynin for treating palmar hyperhidrosis in children younger than 14 years old. Pediatr Dermatol.

[20] Wolosker N, Krutman M, Campdell TPDA, Kauffman P, Campos JRM, Puech-Leão P (2012). Oxybutynin treatment for hyperhidrosis: a comparative analysis between genders. Einstein.

[21] Wolosker N, Teivelis MP, Krutman M (2014). Long-term results of the use of oxybutynin for the treatment of axillary hyperhidrosis. Ann Vasc Surg.

[22] Wolosker N, Teivelis MP, Krutman M (2014). Long-term results of oxybutynin treatment for palmar hyperhidrosis. Clin Auton Res.

[23] Wolosker N, Teivelis MP, Krutman M (2014). Long-term results of oxybutynin use in treating facial hyperhidrosis. An Bras Dermatol.

[24] Wolosker N, Krutman M, Kauffman P, Paula RP, Campos JRM, Puech-Leão P (2013). Effectiveness of oxybutynin for treatment of hyperhidrosis in overweight and obese patients. Rev Assoc Med Bras.

[25] Wolosker N, Teivelis MP, Krutman M (2015). Long-Term efficacy of oxybutynin for palmar and plantar hyperhidrosis in children younger than 14 years. Pediatr Dermatol.

[26] Teivelis MP, Wolosker N, Krutman M, Kauffman P, Campos JRM, Puech-Leão P (2014). Treatment of uncommon sites of focal primary hyperhidrosis: experience with pharmacological therapy using oxybutynin. Clinics (São Paulo).

[27] Karlqvist M, Rosell K, Rystedt A, Hymnelius K, Swartling C (2014). Botulinum toxin B in the treatment of craniofacial hyperhidrosis. J Eur Acad Dermatol Venereol.

[28] Cabreus P, Swartling C, Rystedt A (2019). Postmenopausal craniofacial hyperhidrosis treated with botulinum toxin type B. J Dermatol.

[29] Campos JRM, Kauffman P, Gomes O, Wolosker N (2016). Video-assisted thoracic sympathectomy for hyperhidrosis. Thorac Surg Clin.

[30] Ishy A, Campos JRM, Wolosker N (2011). Objective evaluation of patients with palmar hyperhidrosis submitted to two levels of sympathectomy: T3 and T4. Interact Cardiovasc Thorac Surg.

[31] Lin TS, Wang NP, Huang LC (2001). Pitfalls and complication avoidance associated with transthoracic endoscopic sympathectomy for primary hyperhidrosis (analysis of 2200 cases). Int J Surg Investig.

[32] Katara AN, Domino JP, Cheah W-K, So JB, Ning C, Lomanto D (2007). Comparing T2 and T2-T3 ablation in thoracoscopic sympathectomy for palmar hyperhidrosis: a randomized control trial. Surg Endosc.

[33] Kim DH, Hong YJ, Hwang JJ, Kim KD, Lee DY (2008). Topographical considerations under video-scope guidance in the T3,4 levels sympathetic surgery. Eur J Cardiothorac Surg.

[34] Campos JRM, Wolosker N, Takeda FR (2005). The body mass index and level of resection: predictive factors for compensatory sweating after sympathectomy. Clin Auton Res.

[35] Lin TS, Fang HY (2000). Transthoracic endoscopic sympathectomy for craniofacial hyperhidrosis: analysis of 46 cases. J Laparoendosc Adv Surg Tech A.

[36] Campos JRM, Kauffman P, Wolosker N (2006). Axillary hyperhidrosis: T3/T4 versus T4 thoracic sympathectomy in a series of 276 cases. J Laparoendosc Adv Surg Tech A.

[37] Yazbek G, Wolosker N, Campos JRM, Kauffman P, Ishy A, Puech-Leão P (2005). Palmar hyperhidrosis—which is the best level of denervation using video-assisted thoracoscopic sympathectomy: T2 or T3 ganglion?. J Vasc Surg.

[38] Teivelis MP, Varella AY, Wolosker N (2014). Expanded level of sympathectomy and incidence or severity of compensatory hyperhidrosis. J Thorac Cardiovasc Surg.

[39] Ramsaroop L, Singh B, Moodley J, Partab P, Satyapal KS (2004). Anatomical basis for a successful upper limb sympathectomy in the thoracoscopic era. Clin Anat.

[40] Chung I-H, Oh C-S, Koh K-S, Kim H-J, Paik HC, Lee DY (2002). Anatomic variations of the T2 nerve root (including the nerve of Kuntz) and their implications for sympathectomy. J Thorac Cardiovasc Surg.

[41] Leão LEV, Oliveira R, Szulc R, Mari JJ, Crotti PLR, Gonçalves JJS (2003). Role of video-assisted thoracoscopic sympathectomy in the treatment of primary hyperhidrosis. Sao Paulo Med J.

[42] Andrade LO, Kuzniec S, Wolosker N, Yazbek G, Kauffman P, Campos JRM (2013). Technical difficulties and complications of sympathectomy in the treatment of hyperhidrosis: an analysis of 1731 cases. Ann Vasc Surg.

[43] Wolosker N, Yazbek G, Campos JRM (2010). Quality of life before surgery is a predictive factor for satisfaction among patients undergoing sympathectomy to treat hyperhidrosis. J Vasc Surg.

[44] Campos JRM, Kauffman P, Werebe E C (2003). Questionnaire of quality of life in patients with primary hyperhidrosis. Jornal de Pneumologia. Sociedade Brasileira de Pneumologia e Tisiologia.

[45] Campos JRM, Kauffman P, Werebe EC (2003). Quality of life, before and after thoracic sympathectomy: report on 378 operated patients. Ann Thorac Surg.

[46] Hsu C-P, Shia SE, Hsia JY, Chuang CY, Chen CY (2001). Experiences in Thoracoscopic Sympathectomy for Axillary Hyperhidrosis and Osmidrosis. Arch Surg.

[47] Lehrer E, Nogues A, Jaume F, Mullol J, Alobid I (2020). Assessment of craniofacial hyperhidrosis and flushing by sphenopalatine blockade: a randomized trial. Rhinology.

